# Epigenetic down regulation of G protein-coupled estrogen receptor (GPER) functions as a tumor suppressor in colorectal cancer

**DOI:** 10.1186/s12943-017-0654-3

**Published:** 2017-05-05

**Authors:** Qiao Liu, Zhuojia Chen, Guanmin Jiang, Yan Zhou, Xiangling Yang, Hongbin Huang, Huanliang Liu, Jun Du, Hongsheng Wang

**Affiliations:** 1 Department of Microbial and Biochemical Pharmacy, School of Pharmaceutical Sciences, Sun Yat-sen University, Guangzhou, 510006 China; 2 Sun Yat-sen University Cancer Center, State Key Laboratory of Oncology in South China; Collaborative Innovation Center for Cancer Medicine, Guangzhou, 510060 China; 3 Hunan Cancer Hospital & The Affiliated Cancer Hospital of Xiangya School of Medicine, Central South University, Changsha, 410013 China; 4 Guangdong Institute of Gastroenterology and the Sixth Affiliated Hospital, Institute of Human Virology, Key Laboratory of Tropical Disease Control (Ministry of Education), Sun Yat-sen University, Guangzhou, 510655 China

**Keywords:** GPER, G-1, CRC, NF-κB, ROS

## Abstract

**Background:**

Estrogenic signals are suggested to have protection roles in the development of colorectal cancer (CRC). The G protein-coupled estrogen receptor (GPER) has been reported to mediate non-genomic effects of estrogen in hormone related cancers except CRC. Its expression and functions in CRC were investigated.

**Methods:**

The expression of GPER and its associations with clinicopathological features were examined. The mechanisms were further investigated using cells, mouse xenograft models, and clinical human samples.

**Results:**

GPER was significantly (*p* < 0.01) down regulated in CRC tissues compared with their matched adjacent normal tissues in our two cohorts and three independent investigations from Oncomine database. Patients whose tumors expressing less (*n* = 36) GPER showed significant (*p* < 0.01) poorer survival rate as compared with those with greater levels of GPER (*n* = 54). Promoter methylation and histone H3 deacetylation were involved in the down regulation of GPER in CRC cell lines and clinical tissues. Activation of GPER by its specific agonist G-1 inhibited proliferation, induced cell cycle arrest, mitochondrial-related apoptosis and endoplasmic reticulum (ER) stress of CRC cells. The upregulation of reactive oxygen species (ROS) induced sustained ERK1/2 activation participated in G-1 induced cell growth arrest. Further, G-1 can inhibit the phosphorylation, nuclear localization, and transcriptional activities of NF-κB via both canonical IKKα/ IκBα pathways and phosphorylation of GSK-3β. Xenograft model based on HCT-116 cells confirmed that G-1 can suppress the in vivo progression of CRC.

**Conclusions:**

Epigenetic down regulation of GPER acts as a tumor suppressor in colorectal cancer and its specific activation might be a potential approach for CRC treatment.

**Electronic supplementary material:**

The online version of this article (doi:10.1186/s12943-017-0654-3) contains supplementary material, which is available to authorized users.

## Background

Colorectal cancer (CRC), also called colon cancer or large bowel cancer, is the second most common cause of cancer death and accounts for almost 10% of all reported cancer cases in the world [1]. Given the high incidence in the aging population and high mortality rates of CR, new prevention strategies are needed. Clinical data revealed that the incidence of colon cancer is significantly (*p* < 0.05) lower in women than in men, which may be due to the presence of estrogens [2]. Postmenopausal women receiving combined hormone replacement therapy will significantly reduce the risk of colorectal cancer [3, 4]. Further, young women (18–44 years old) of colorectal cancer have a better overall survival compared with men of the same age [5]. However, this protection is lost when a woman reaches menopause. Cellular and animal studies also suggested role of estrogens in the reduction of colon cancer occurrence [6, 7]. These data suggested that estrogenic signals might be involved and have protection roles in the development of this disease.

The mechanism behind this observed protection, however, is poorly understood. The classical activation of estrogen is mainly mediated via the two nuclear estrogen receptors (ER α/β). Several studies indicated the suppressive effect of ERβ in the progression of colon cancer. The expression of ERβ is correlated to prognosis of colon cancer, while its decrease expression is found in higher grade and larger tumors [8]. The loss of expression of ER β is inversely correlated with more advanced Dukes' staging in colon cancer [9]. Cellular experiments supported the tumor suppressive effect of ER β in colon [10]. From the phenotype of ER β^−/−^ mice, there is more colitis-associated neoplasia [11] and ERβ in the colon appears to decrease proliferation and increase apoptosis [12]. All these data suggested that ERβ is important for the tumor suppressive effect, while ERα is not widely expressed and its role in colon cancer has not been found so far [10].

Recently, G-protein coupled estrogen receptor (GPER), a member of G-protein coupled receptors (GPCRs), has been shown to mediate rapid non-genomic estrogenic effects of estrogen, phytoestrogen, and xenoestrogen [13]. The activation of GPER can stimulate its downstream signals including mitogen-activated protein kinase (MAPK), phosphoinositide 3-kinase (PI3K), and epidermal growth factor receptors (EGFRs) [14], then modulate growth of hormonally responsive cancers such as endometrial [15], ovarian [16], and breast cancer [17]. Our recent studies revealed that activation of GPER by its specific agonist G-1 can suppress the proliferation and migration of ER negative breast cancer cells [18]. Although epidemiological and experimental data reveal estrogenic signals are important for the progression of CRC, the expression and roles of GPER in CRC have not been studied yet.

In the present study, we demonstrated that expression of GPER is obviously decreased in CRC tissues and cell lines due to promoter methylation and histone deacetylation. Activation of GPER can inhibit proliferation, induce G2/M cell cycle arrest, mitochondrial-related apoptosis, and endoplasmic reticulum (ER) stress of colon cancer cells. The suppression of NF-κB pathways and activation of ROS/MAPK signals participated G-1 induced growth arrest. To our knowledge, this is the first study to investigate the expression and effects of GPER, an important mediator of non-genomic estrogenic effects, on the progression of CRC. The presented data provide novel insights into the estrogenic signals on the progression of CRC and suggest that target activation of GPER is beneficial for CRC treatment.

## Methods

### Tissue samples and GPER expression analysis

This study enrolled two independent cohorts of CRC patients. A group of 32 clinical-pathological (Cohort 1) characterized patients with histologically confirmed CRC from the Sixth Affiliated Hospital of Sun Yat-sen University between 2008 and 2014, the detailed information was summarized in (Additional file 1: Table S1). All tissue samples were selected by an experienced pathologist immediately after surgical resection, snap frozen in liquid nitrogen, and then stored at −80 °C. The mRNA expression of GPER in Cohort 1was measured by real-time PCR. The other cohort containing 90 CRC and 90 paired non-CRC counterparts was from the tissue microarray (HCol-Ade180Sur-06) provided by Outdo Biotech Co., Ltd. (Shanghai, China). All tissues were collected from 2006 to 2007, and the follow up data were acquired in the next 7-8 years. Detailed clinicopathological features were listed in Table 1. These tumor tissues and the adjacent normal tissues were IHC stained for GPER and quantified. In addition, the expression of GPER in CRC tissues was further obtained from the Oncomine Database (www.oncomine.org) as follows: Skrzypczak colorectal 1 and 2, Sabates-Bellver Colon [19], and TCGA colorectral. The sample information and expression data were available in the Gene Expression Omnibus (GEO) database [Accession nos. GSE2091 (Skrzypczak colorectal 1 and 2), GSE871 (Sabates-Bellver Colon), and A_23_P8631 (TCGA Colorectal) at https://www.ncbi.nlm.nih.gov/geo/].

**Table 1:** The detailed clinicopathological features of clinical tissues (chort2)

Characteristics		N	Mean	*p* value
Tumor/Adjacent	Tumor	90	6.56 ± 4.27	0.038
	Adjacent	90	7.71 ± 3.03	
Age	≤60	18	7.56 ± 4.2	0.270
	>60	72	6.56 ± 4.28	
Sex	male	47	6.66 ± 4.10	0.811
	female	43	6.44 ± 4.50	
Tumor volume	≤50 cm^3^	53	6.21 ± 4.08	0.489
	>50 cm^3^	35	6.86 ± 4 .59	
Stage	1	7	10.4 ± 1.99	0.018
	2	47	6.76 ± 4.19	
	3	32	5.43 ± 4.07	
	4	2	0.50 ± 0.71	
Tumor (T)	T1	3	8.33 ± 6.35	0.169
	T2	6	8.16 ± 4.40	
	T3	68	6.46 ± 4.31	
	T4	11	4.81 ± 2.89	
Lymph Node (N)	N0	56	7.41 ± 4.17	0.037
	N1	25	6.11 ± 4.34	
	N2	9	4.80 ± 4.07	

### Cell culture and reagents

Colon cancer cell lines HCT-116, LS147T, SW620, HCT15, HCT8, SW480, HT29 and human colon mucosal epithelial NCM460 cells were obtained from American Type Culture Collection (Manassas, VA) and cultured in RPMI 1640 or DMEM medium with 10% fetal bovine serum and 1% penicillin-streptomycin (PEST) (Invitrogen) at 37 °C with 5% CO_2_ atmosphere. An ABI 3130 Genetic Analyzer (Applied Biosystems) was used for the profiling. The DNA profile data were cross-checked with the ATCC data bank. The purified rabbit antibody against GPER (SP4677P) for IHC was purchased from Acris antibodies (Herford, Germany), for western blot was from Santa Cruz Biotech (sc-48524-R, Heidelberg, Germany). Other antibodies for western blot assay were purchased from Cell Signaling Technology Inc. (Beverly, MA, USA) excluding antibodies against p-Akt and Akt, which were purchased from Bioworld Technology, Inc (Minneapolis, MN, USA). GPER agonist G-1, inhibitors, and other chemicals were of reagent grade or better and purchased from Sigma Chemical Co. (St. Louis, MO, USA) unless otherwise noted. All compounds were solubilized in DMSO. Medium containing 0.5% DMSO was used as the control.

### RNA-extraction and real-time PCR

RNA extraction with Trizol (Invitrogen) and real time PCR were done according to the protocol used in our previous study [18]. The primers of targeted genes were as follow: GAPDH, forward 5′-GCA CCG TCA AGG CTG AGA AC-3’ and reverse 5′-TGG TGA AGA CGC CAG TGG A-3′; GPER, forward 5′-CGT CAT TCC AGA CAG CAC CGA G-3′ and reverse 5′-CGA GGA GCC AGA AGC CAC ATC-3′; IKKα, forward 5′- GCC ATC CAC TAT GCT GAG GT-3′ and reverse 5′- CGC TGT TCC AGA GAT TCC AT-3′; IKKβ, forward 5′- TTC TTC AAA ACC AGC ATC CA-3′ and reverse 5′-GAG CCA TCA TCC GTT CTA CC-3′; IKKγ, forward 5′- GAC CCC GCA GAC TAT CAA TC-3′ and reverse 5′-CGC CTG GAA CAG CAT CTT-3′. GAPDH was used as a control for normalization.

### Western blot analysis and immunoprecipitation

Western blot analysis and immunoprecipitation were performed as previously described [20] and described in detail in the Additional file 1.

### Bisulfite genomic DNA sequencing

To analyze the methylation of GPER promoter, genomic DNA of CRC cells was prepared using TIANamp Genomic DNA kit (TIANGEN), followed by the treatment of sodium bisulfite using the Epitect Bisulfite DNA kit (QIAGEN, cat: 59824). Products were amplified by PCR primer pairs used to recognize the bisulfite-modified regions (-781 to -461) of the GPER promoter as: forward 5’- TTG AAG TTT TTT TTT GAG GAA-3’, reverse 5’- TAA TAA CCT CTT CCC CACC-3’.

### Chromatin immunoprecipitation (ChIP)

ChIP was executed using the EpiQuik Acetyl-Histone H3 chip kit (Epigentek Group Inc, NY) for the cell line and clinical samples according to the manufacturer’s protocol and previous study [21]. Briefly, we sonicated the crosslinked chromatin DNA into 200- to 1000-bp fragments. The chromatin was immunoprecipitated using an anti-acetyl-histone H3 antibody. Normal mouse IgG was used as the negative control for validating the ChIP assay. Quantitative RT-PCR was conducted using SYBR Green Mix (Takara Bio) with the primers specific for the GPER promoter: forward, 5’- ATT TCC CAA AAC AAT GAC CCC TT-3’ and reverse, 5’- AGA AGT TCA GCG GTT TCC TCA-3’.

### Cell viability assay

Cell viability was detected by use of CCK-8 kit (Dojindo Molecular Technologies, Gaithers burg, MD, USA) according to previously described procedures [22]. All experiments were performed in duplicates. The 50% inhibitory concentration (IC_50_) was calculated using SPSS 17.0 for Windows.

### Flow cytometry

Flow cytometry was used to analyze cell cycle, apoptosis, mitochondrial membrane potential (∆Ψm), and reactive oxygen species (ROS) affected by G-1 treatment as our previous methods [18]. The detailed procedures were stated in Additional file 1.

### Immunofluorescence

Immunofluorescent staining was carried out as described previously [18] and described in detail in Additional file 1.

### Luciferase reporter assay

Luciferase activity was measured using the Dual Luciferase Reporter Assay kit (Promega) according to the manufacturer’s instructions. In brief, cells at approximately 70% confluence were transfected with 0.2 μg DNA/cm^2^ of pNF-kB-luc plasmid and lipofectamine 2000 reagent (Invitrogen, USA) according to the manufacturer’s instructions, and then treated with G-1 for the indicated times. Then cells were lysed and luciferaseactivity was measured using a dual-luciferase assay kit (Promega). pRL-TK was co-transfected as a control.

### Experimental animals and xenograft models

Female nude mice (four weeks old, *n* = 8 for each group) were purchased from the Sun Yat-sen University (Guangzhou, China) Animal Center and raised under pathogen-free conditions. All animal studies were conducted in accordance with institutional guidelines for the care and use of experimental animals. HCT-116 colon cancer cells (2 × 10^6^ per mouse) were diluted in 200 μL normal medium + 200 μL Matrigel (BD Biosciences) and injected into immunodeficient mice to investigate tumor growth. When the tumor grows to 100 mm^3^, mice of G-1 group were treated with G-1 (2 mg per kg, body weight) by intratumoral injection for four times for every three days. Control group was treated with an equal volume of vehicle. Tumor growth and body weight were monitored every three days. The tumor volume was calculated using the formula V = 1/2× larger diameter × (smaller diameter) ^2^. At the end of treatment, the animals were sacrificed, and the tumors were removed and weighed for use in histology analysis.

### Immunohistochemistry (IHC)

Immunohistochemistry was performed to measure the expression of Ki-67, p65, p-ERK1/2, and p27. Tumor tissues were fixed in formalin and embedded in paraffin. Sections (5 μm) were cut and stained with H&E. For immunohistochemical staining, sections were deparaffinized and hydrated, and endogenous peroxidase activity was blocked with 3% H_2_O_2_ in water for 10 min. Antigen retrieval was done with 10 mM citrate buffer (pH 6.0) for 10 min. Slides were blocked with Biocare reagent for 10 min and then incubated with primary antibodies overnight at 4 °C. After washed in PBS twice, slides were incubated with goat anti-rabbit horseradish peroxidase-conjugated secondary antibodies for 30 min at room temperature and then washed. Finally, slides were incubated with 3, 3’-diami-nobenzidine and counter stained with hematoxylin. The clinical slides of GPER were analyzed separately by two pathologists without knowing the patients’ clinical information. The staining intensity was scored according to previous study [23]. The intensity was scored on a scale of 0–3 as negative (0), weak (1), medium (2) or strong (3). The extent of the staining, defined as the percentage of positive stained areas of tumor cells per the whole tumor area, was scored on a scale of 0 (0%), 1 (1–25%), 2 (26–50%), 3 (51–75%) and 4 (76–100%). An overall protein expression score (overall score range, 0 to 12) was calculated by multiplying the intensity and positivity scores. For statistical purposes the staining score was further categorized as low (0–5), medium (6–8), and high (9–12).

### Statistical analyses

The statistical analyses were performed using SPSS 17.0 for Windows. A p-value of < 0.05 was considered to be statistically significant. Data were analyzed by two-tailed unpaired Student's t-test between two groups. For multiple comparisons, One-Way ANOVA was used followed by Bonferroni test.

## Results

### GPER expression was down regulated in CRC tissues

**Fig. 1:**
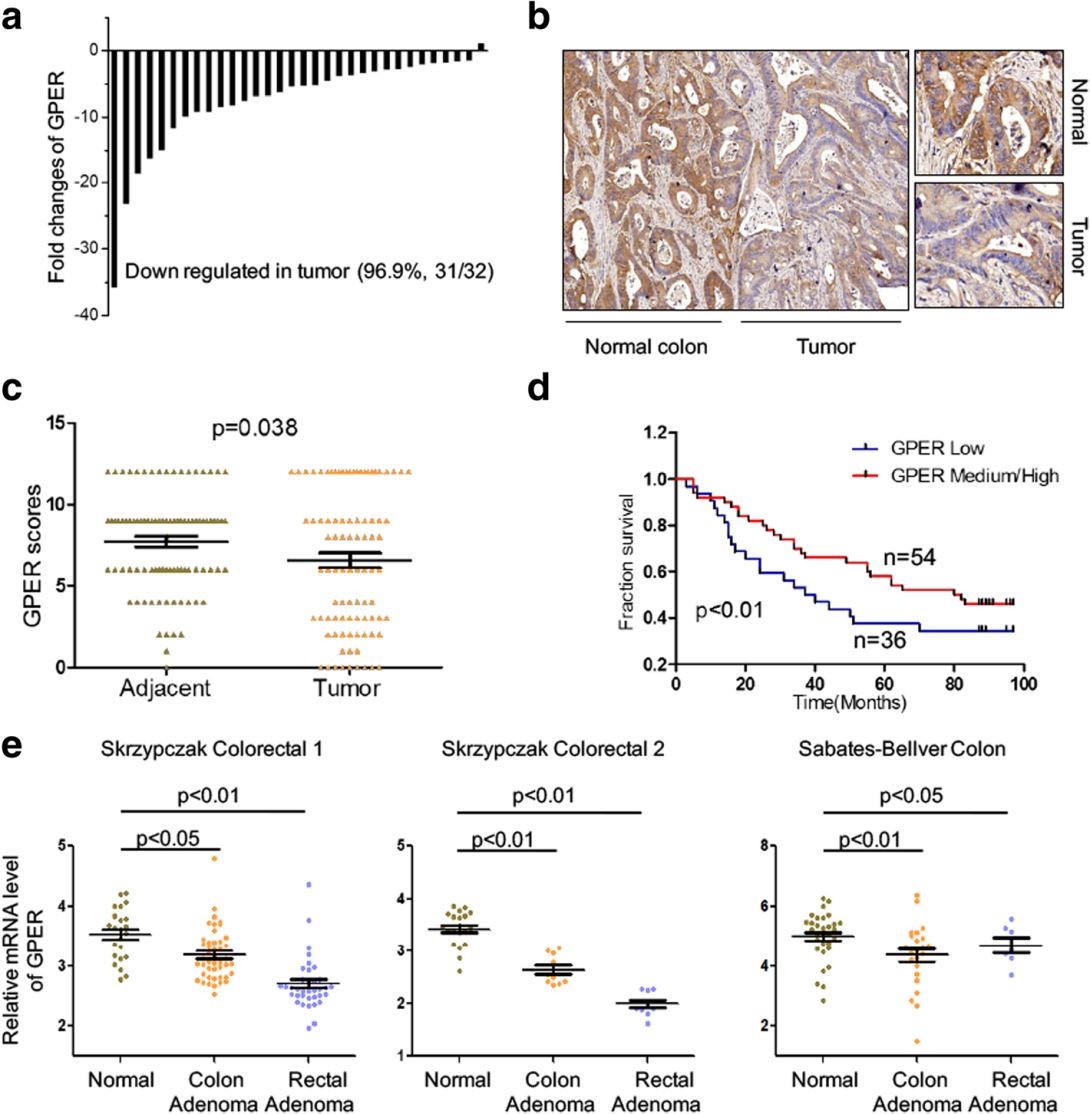
GPER expression was down regulated in CRC tissues. **a** GPER expression was examined by quantitative RT-PCR in 32 paired human colon cancer tissues and adjacent normal mucosa tissues (Cohort 1); **b** Typical immunohistochemical staining for GPER expression in a tumor and its adjacent tissue from a CRC patient in the commercial tissue microarray (Cohort 2, left 10 X, right 40 X); **c** Scores for GPER staining in tumor and adjacent normal tissue samples from CRC patients in the commercial tissue microarray (Cohort 2); **d** Overall survival (OS) in patients with high/medium levels of GPER (*n* = 54) vs the remaining patients (*n* = 36) was plotted by the Kaplan-Meier method; **e** The relative mRNA expression of GPER in three Oncomine datasets: Skrzypczak Colorectal 1, Skrzypczak Colorectal 2, and Sabates-Bellver Colon

To explore the role of GPER in the progression of CRC, we compared mRNA levels of GPER between CRC tissues and paired adjacent non-cancerous mucosa from 32 individual patients (Cohort 1). GPER was successfully amplified in all tumor and normal specimens analyzed. According to qRT-PCR analysis, GPER expression was significantly decreased in 31 of 32 (96.9%) tumor samples, compared with the adjacent normal mucosa tissues (Fig. 1a). The mRNA expression of GPER in tumor tissues was 7.7 fold less than that in adjacent normal mucosa tissues in the present study. However, there was no significant difference for the GPER expression among different age, gender or stages (Additional file 1: Table S1), which might be due to the small sample size. The protein expression of GPER was further evaluated in tissue microarrays containing 90 samples of CRCs with patient-matched normal mucosal tissues (Cohort 2). IHC analyses demonstrated significantly (*p* < 0.05) decreased expression of GPER in CRC tumor samples compared with adjacent normal mucosal tissues (Fig. 1 b & c, Table 1). Moreover, log-rank statistical test suggested that patients whose tumors expressing less GPER (*n* = 36) showed poorer survival rate as compared with those with greater levels (*n* = 54) of GPER (*p* < 0.01) (Fig. 1d). As summarized in Table 1, the expression of GPER was significantly (*p* < 0.05) decreased with the increasing of stage and lymph node metastasis of CRC patients (Table 1), suggesting that loss of GPER might be an early event of CRC progression. In addition, GPER expression decreased in CRC patients with higher grade of tumor (T) with a marginal statistical significance of *p* = 0.169. There was no significant variation of GPER expression among different age and sex (Table 1). Furthermore, reanalysis of GPER expression in the CRC Affymetrix datasets published by Skrzypczak et al. [19] produced similar results (Fig. 1e). We further analyzed the expression of GPER in 237 samples from TCGA Colorectal data bases. The results showed that the mRNA levels of GPER in males (*n* = 117) were significantly (*p* < 0.01) less than that in females (*n* = 120) (Additional file 1: Figure S1). Collectively, our present study and published data revealed that GPER is significantly down regulated in the CRC tissues as compared with the adjacent normal tissues and higher expression of GPER is correlated with favorable prognosis of CRC patients.

### Promoter methylation and histone H3 deacetylation were involved in GPER down regulation in CRC cell and tissues

**Fig. 2:**
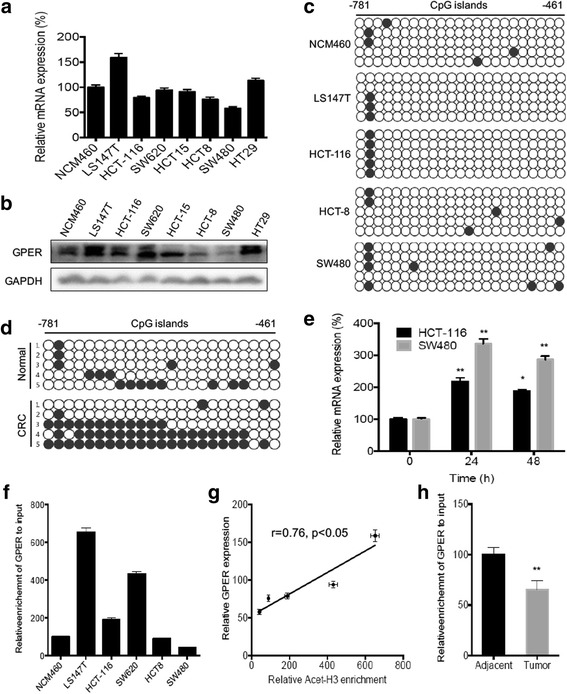
Promoter methylation and histone H3 deacetylation were involved in GPER down regulation in CRC cell and tissues. The mRNA (**a**) and protein (**b**) levels of GPER in CRC cell lines, human colon mucosal epithelial NCM460 cells were measured by qRT-PCR and western blot analysis, respectively; (**c**) Methylation status of GPER promoter in CRC cell lines was determined by bisulfite genomic DNA sequencing. Each dot represents a CpG site. White dots represent unmethylated CpG dinucleotides whereas each black dots represents a methylated cytosine residue within the CpG islands; (**d**) Methylation statuses of GPER promoter in five pairs of CRCs tissues and patient-matched normal tissues (Cohort 1) were determined by bisulfite genomic DNA sequencing; (**e**) HCT-116 or SW480 cells were treated with 5 μM 5-Aza for the different times, then mRNA of GPER was measured by use of qRT-PCR; (**f**) ChIP analysis of NCM460 and CRC cell lines were conducted on the GPER promoter regions by use of antiacetyl histone H3 antibody; (**g**) The correlation between relative acet-H3 enrichment and GPER mRNA expression in LS147T, HCT-116, SW620, HCT8, and SW480 cells; (**h**) ChIP analysis of five pairs of CRCs tissues and patient-matched normal tissues (Cohort 1) were conducted on the GPER promoter regions by use of antiacetyl histone H3 antibody. Data were presented as means ± SD of three independent experiments. *p < 0.05 compared with control; **p < 0.01 compared with control

GPER expression was detected in the colon cancer cell lines LS147T, HCT-116, SW620, HCT-15, HCT-8, SW480, HT29, and human colon mucosal epithelial NCM460 cells (Fig. 2a & b). These results suggested that GPER is down regulated in colorectal cancer cells such as HCT-8 and SW480, while up regulated in LS147T. Since the expression of GPER showed a diverse level, we divided these cell lines into high (LS147T), middle (NCM460, HCT-116), and low (HCT-8 and SW480) groups and analyzed the potential role of DNA methylation on GPER promoter. A total of 23 CpG sites located between nucleotides -781 and -461 in the GPER promoter were examined using bisulfite genomic DNA sequencing. The results showed that low GPER group (SW480 and HCT-8) had obvious greater methylation level comparing with high GPER group (LS147T) (Fig. 2c). Similar results were also observed in methylation statuses of GPER promoter in clinical samples. We found that the methylation of GPER promoter in 5 CRC tissues were significantly greater than that in patient-matched normal tissues (Fig. 2d). This was further confirmed by the in vitro results that 5-aza-dC (a DNA methyltransferase inhibitor) can significantly (*p* < 0.05) increased the mRNA expression of GPER in both HCT-116 and SW480 cells (Fig. 2e). However, the expression of GPER in these cell lines did not correspond with their tumorigenic or metastatic potentials, which might be due to that other factors such as disease, gender and/or age of source patients can also regulate the expression of GPER.

We further observed that up regulation of GPER in LS147T and SW620 was associated with increased histone H3 acetylation across GPER promoter region when compared with the human colon mucosal epithelial NCM460 cells. Consistently, the down regulation of GPER in HCT-8 and SW480 cells were associated with decreased histone H3 acetylation (Fig. 2f). In addition, the significantly positive correlation (*r* = 0.76, *p* < 0.05) was observed between relative histone H3 acetylation and GPER mRNA expression among the five detected CRC cell lines (Fig. 2g). This was further confirmed by the clinical results that histone H3 acetylation of GPER promoter was significantly (*p* < 0.01) decreased in 5 CRC tissues as compared with patient-matched normal tissues (Fig. 2h). Collectively, our data revealed that promoter methylation and histone H3 deacetylation were involved in the down regulation of GPER in CRC cell lines and tissues.

### Targeted activation of GPER inhibited in vitro growth of CRC lines

**Fig. 3:**
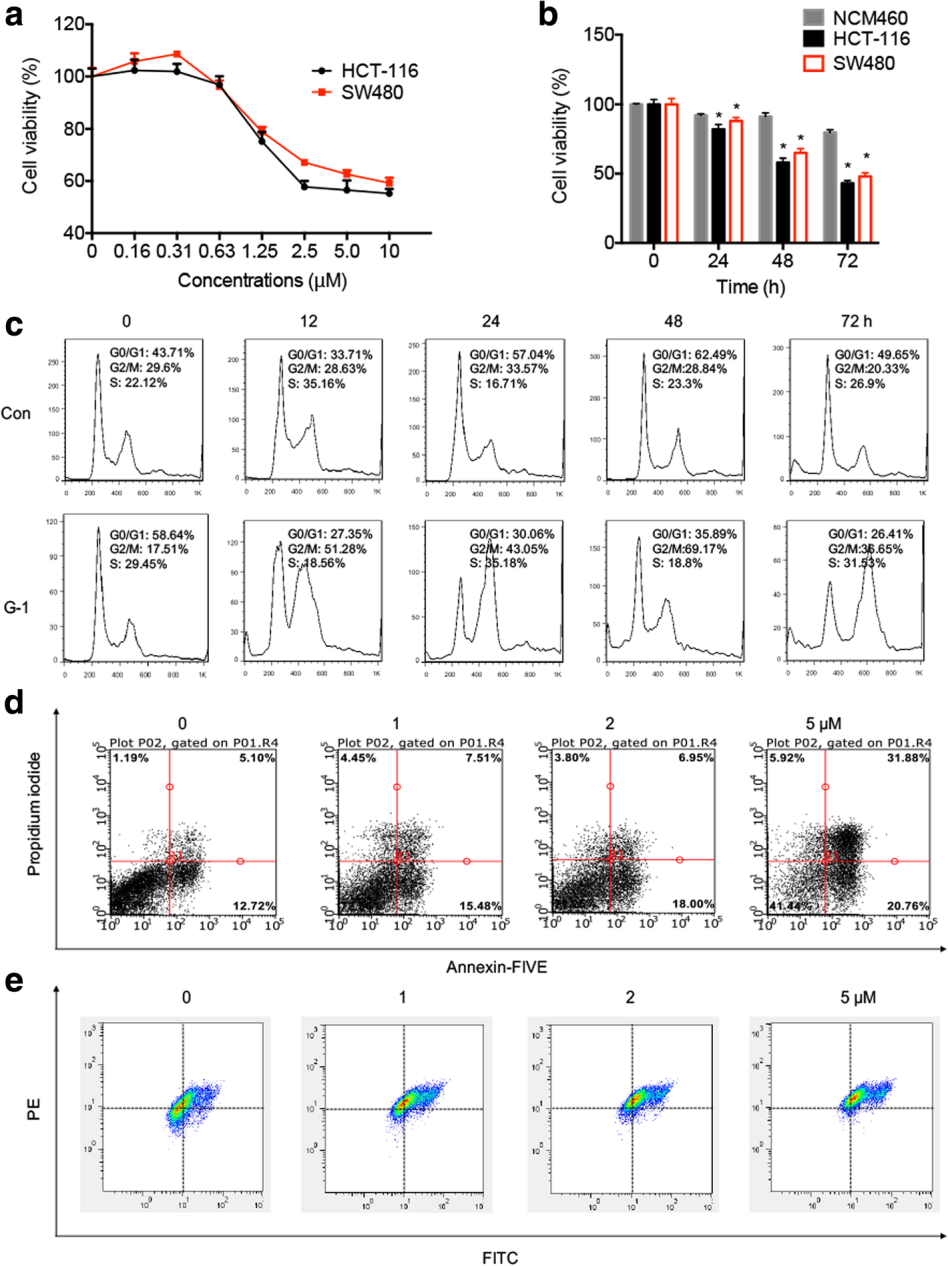
Targeted activation of GPER inhibited in vitro growth of CRC cells. Cells were treated with various concentrations of G-1 for 48 h (**a**) or 1 μM G-1 for the indicated times (**b**), then cell viability was assessed by CCK-8 kit; (**c**) HCT-116 cells were synchronized at the G1/S transition by a double TdR block, and then treated with 1 μM G-1 for the indicated times. The cell cycles were analyzed by FCM; (**d**) HCT-116 cells were treated with increasing concentrations of G-1 for 48 h, stained with annexin V-FITC and PI, and then analyzed by FCM for cell apoptosis; (**e**) HCT-116 cells were treated with G-1 as the indicated concentrations for 24 h, and then JC-1, the mitochondria-specific dye, was added to measure the membrane polarity (ΔΨ*m*) and cell apoptosis. Apoptotic cells mainly show green fluorescence (FITC), while healthy cells show red fluorescence (PE). Data were presented as means ± SD of three independent experiments. *p < 0.05 compared with control

To extend the clinical studies, we next aimed to investigate roles of GPER in growth regulation of colon cancer cells. Two CRC cell lines HCT-116 and SW480 were treated with G-1 (GPR30 specific agonist) to study the activation of GPER on cell proliferation. We found that G-1 significantly inhibited the proliferation of both HCT-116 and SW480 cells via concentration and time independent manners (Fig. 3a&b). The IC_50_ values of G-1 (48 h) to HCT-116 and SW480 cells were 8.55 and 11.7 μM, respectively. Therefore 1 μM G-1 was chose for further studies of GPER activation on the proliferation of CRC cells on the basis of cytotoxicity test and other previous studies [18, 24, 25].

We then synchronized cells using double TdR-blocking method so that cells can stay in a same stage. Flow cytometric analysis showed a significant (*p* < 0.05) increase in the number of cells in G2/M phase after treatment of HCT-116 cells with G-1 for 12 h, as compared with that in DMSO (0.5%, v/v) treated control cells. The increase of G2/M phases by G-1 lasted throughout 72 h treatment period (Fig. 3c), indicating induction of a persistent cell-cycle arrest in the G2/M stage of the cell cycle by G-1 in HCT-116 cells. Similar G2/M arrest by G-1 was also observed in SW480 cells (Data not shown). These data supported the notion that G-1 suppressed CRC cell proliferation via accumulating the cells in G2/M.

The cell cycle analysis suggested that G-1 treatment can increase the proportion of cells at apoptotic sub-G1 phase (Fig. 3c). Then we investigated the effects of G-1 on cellular apoptosis by FCM. Our results showed that G-1 treatment resulted in a marked dose dependent increase in apoptosis of both HCT-116 (Fig. 3d) and SW480 (Additional file 1: Figure S2A) cells. Further, the mitochondrial membrane potential (ΔΨ*m*) was measured using fluorochrome dye JC-1. Our results showed that G-1 treatment resulted in a concentration dependent significant increase in the ration of the green fluorescence to red fluorescence (Fig. 3e), suggesting that activation of GPER can decrease the ΔΨ*m* and then promote the cell apoptosis. The expression levels of apoptotic related proteins in HCT-116 cells treated with G-1 for 48 h were further measured. As shown in Additional file 1: Figure S2B, activation of GPER obviously up regulated the expression of Bax, p21, and cleaved caspase-3, while down regulated the expression of Bcl-2 and procaspase-3. In addition to mitochondrial pathway, ER stress also plays an important role in cancer cell growth arrest and apoptosis [26, 27]. Our study further revealed that G-1 treatment can increased the expression of ER stress-related proteins including transcription factor 4 (ATF4), transcription factor 6 (ATF6), X-box binding proteins 1 (XBP-1), and C/EBP-homologous protein (CHOP) via a time dependent manner in HCT-116 cells (Additional file 1: Figure S2C). Collectively, our results revealed that activation of GPER induced mitochondrial-related apoptosis and ER stress in CRC cells.

### ROS/ERK1/2 signals were involved in suppression effects of G-1 on cell growth

**Fig. 4:**
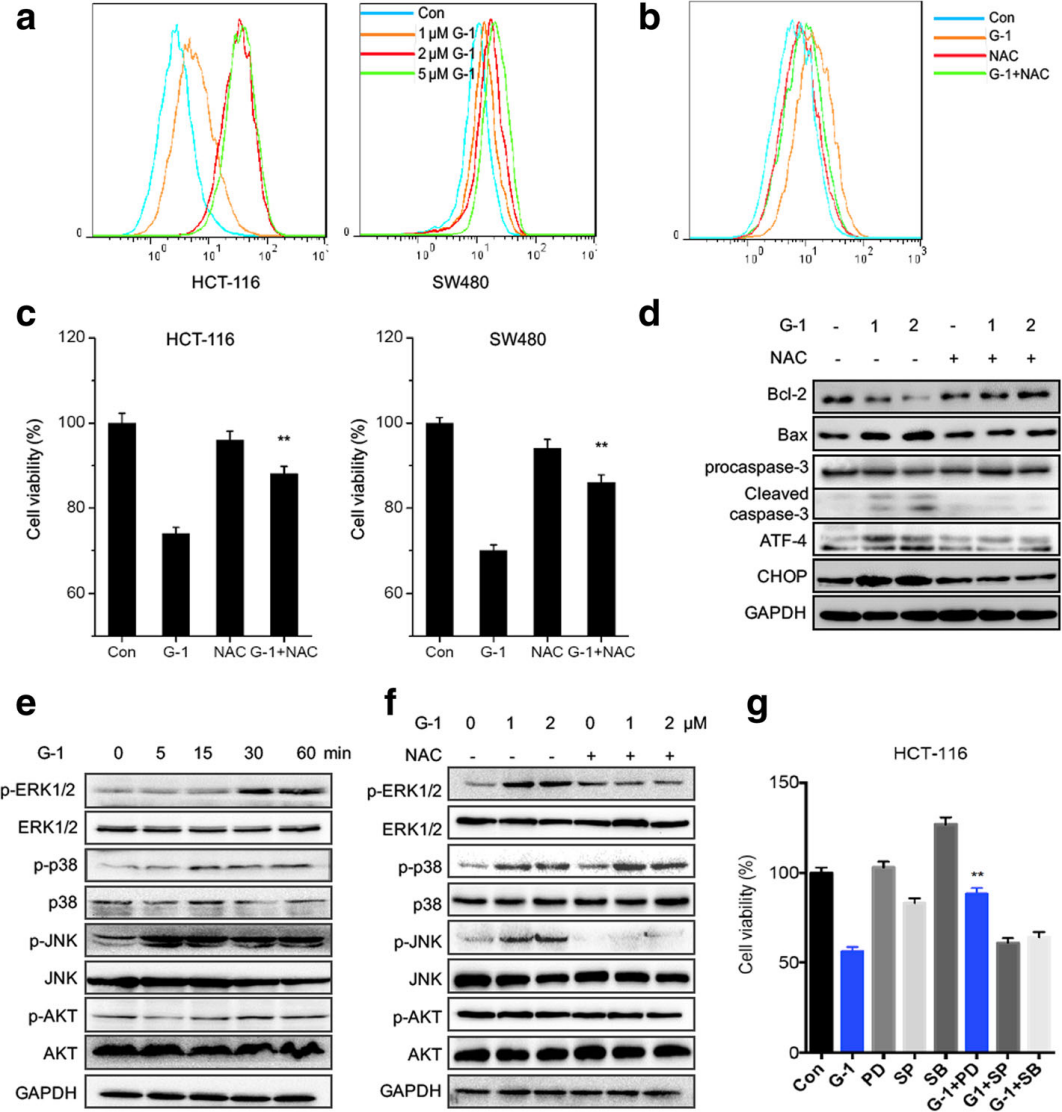
ROS/ERK1/2 signals were involved in suppression effects of G-1 on CRC cell growth. (**a**) HCT-116 and SW480 cells were treated with various concentrations of G-1 for 3 h, and then loaded with CM-H2DCFDA. The fluorescence intensity was measured by FCM; (**b**) HCT-116 cells were pretreated with NAC (20 mM) for 1 h and then treated with 1 μM G-1 for 3 h, and then loaded with CM-H2DCFDA; (**c**) HCT-116 or SW480 cells were pretreated with NAC (20 mM) for 1 h and then treated with 1 μM G-1 for 48 h, cell viability was assessed by CCK-8 kit; (**d**) HCT-116 cells were treated with indicated concentrations of G-1 for 48 h with or without NAC pretreated for 1 h, and the protein expression was examined by western blot analysis; (**e**) HCT-116 cells were treated with 1 μM G-1 for 0 to 60 min, the total and phosphorylation of MAPK and Akt were measured by western blot analysis; (**f**) HCT-116 cells were pretreated with NAC (20 mM) for 1 h and then treated with 1 μM G-1 for 30 min, the protein expression was examined by western blot analysis; (**g**) HCT-116 cells were pretreated with 10 μM ERK1/2 inhibitor PD98059(PD), JNK inhibitor SP600125 (SP), or p38-MAPK inhibitor SB203580 (SB), and then exposed to 1 μM G-1 for 48 h, the cell viability was measured by use of CCK-8 kit. Data were presented as means ± SD of three independent experiments. **p < 0.01 compared with G-1 group

ROS generation plays an important role in growth arrest, mitochondrial-related apoptosis, and ER stress of cancer cells [28]. Then we measured the effects of G-1 on intracellular ROS accumulation by measuring the fluorescent intensity of DCF-DA. Our results revealed that G-1 significantly increased the ROS generation via a dose of dependent manners in both HCT-116 and SW480 cells (Fig. 4a). To investigate the roles of ROS in G-1 induced growth arrest of CRC cells, we attenuated G-1 induced ROS generation by pretreatment cells with ROS scavenger NAC for 1 h (Fig. 4b). The cell viability tests showed that NAC also significantly (*p* < 0.05) alleviated the inhibition effects of G-1 on the proliferation effects of HCT-116 and SW480 cells (Fig. 4c). Further, NAC also markedly attenuated G-1 induced downregulation of Bcl-2 and up regulation of Bax, cleaved caspase-3, ATF-4, and CHOP (Fig. 4d).

The results suggested that ROS mediated, at least partially, G-1 induced ER stress and growth arrest. The elevated ROS generation can activate its downstream signals including MAPKs and PI3K/Akt [29]. We used total and phosphor-specific antibodies to monitor the expression levels and activation statuses, respectively, of MAPKs and Akt. As shown in Fig. 4e, G-1 treatment can rapidly activate ERK1/2, JNK, and p38-MAPK, while had no obvious effect on the activation of AKT. Further, the activation of ERK1/2 and p38-MAPK also varied in G-1 treated HCT-116 cells via a dose dependent manner (Additional file 1: Figure S3A). The G-1 induced activation of ERK1/2 and p38-MAPK can last more than 12 h, while no similar result observed for JNK (Additional file 1: Figure S3B). To verify whether G-1 induced ROS generation is the inducer of variation of these signals, HCT-116 cells were pretreated with ROS scavenger NAC to block G-1 induced ROS generation. The results showed that NAC significantly attenuated G-1 induced activation of ERK1/2 and JNK, while had no obvious effect on G-1 induced phosphorylation of p38-MAPK (Fig. 4f). To test whether MAPK signals participated in G-1 induced growth arrest of CRC cells, we pretreated HCT-116 cells with their specific inhibitors and then treated with 1 μM G-1 for 48 h to measure cell viability. Our results revealed that the inhibitor of ERK1/2, while not JNK or p38-MAPK, can significantly (*p* < 0.01) attenuated the suppression effects of G-1 on the proliferation of HCT-116 cells (Fig. 4g). These results revealed that ROS/ERK1/2 signal was involved in G-1 induced growth arrest of CRC cells.

### Inhibition of NF-κB participated G-1 induced growth arrest

**Fig. 5:**
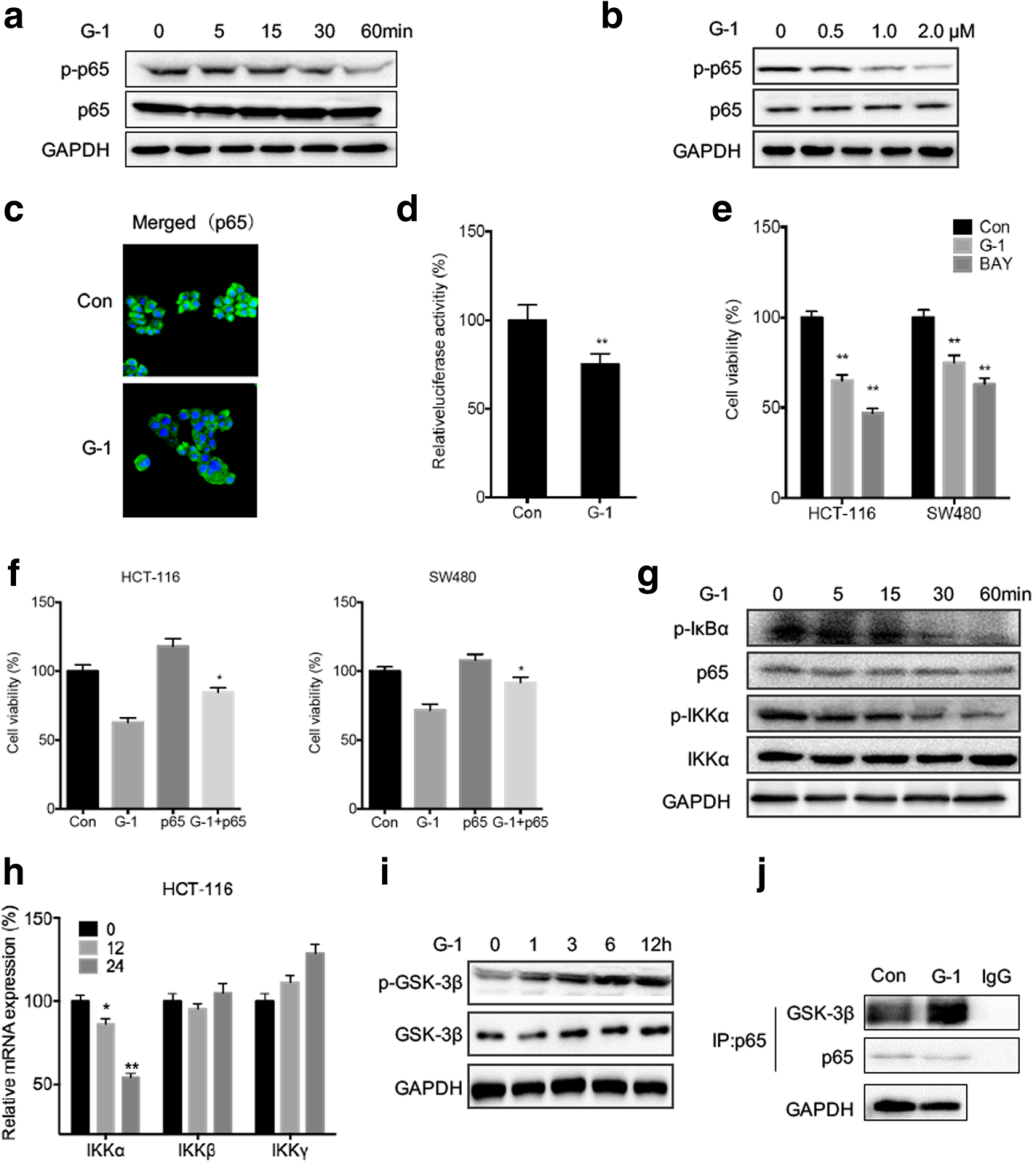
Inhibition of NF-κB participated in G-1 induced growth arrest. HCT-116 cells treated with 1 μM G-1 for 0 to 60 min (**a**) or increasing concentrations of G-1 for 30 min (**b**), the total and phosphorylation of p65 were measured by western blot analysis; (**c**) HCT-116 cells were treated with or without G-1 for 6 h, and the subcellular localization of p65 (green) was examined by immunofluorescence staining and nuclei were stained with DAPI (blue); (**d**) HCT-116 cells were transfected with pGL3-Basic-luc reporter plasmid containing 5 copies of the κB site plasmid and pRL-TK plasmids which served as the correcting transfection efficiency and then treated with or without G-1 (1 μM) for 24 h, then the lysates were assayed. Shown are relative luciferase activities normalized to Renilla activities; (**e**) HCT-116 or SW480 cells were treated with 1 μM G-1 or 10 μM NF-κB inhibitor BAY11-7082 (BAY) for 48 h, the cell viability was measured by use of CCK-8 kit; (**f**) HCT-116 or SW480 cells were transfected with pcDNA3.1 (vector) or pcDNA3.1/p65 for 24 h and then treated with 1 μM G-1 for 48 h, the cell viability was measured by use of CCK-8 kit, *p < 0.05 compared with G-1 group.; (**g**, **h**, & **i**) HCT-116 cells were treated with 1 μM G-1 for the indicated times, proteins were examined by western blot analysis, mRNAs were measured by qRT-PCR; (**j**) HCT-116 cells were treated with G-1 for 12 h, and then p65 was immunoprecipitated from equal amount of lysates and the associated GSK-3β was detected by western blot analysis. Data were presented as means ± SD of three independent experiments. *p < 0.05, **p < 0.01 compared with the control group

The inhibition of NF-κB is involved in G-1 induced suppression of EMT of breast cancer cells [23]. In the CRC cells, our data showed that G-1 treatment can rapidly suppress the phosphorylation of p65 via both time (Fig. 5a) and dose (Fig. 5b) dependent manners. In addition, G-1 also significantly decreased the nuclear translocation of p65 (Fig. 5c) and reduced the transcriptional activity of pGL3-Basic-NF-κB-luc in SW480 cells (Fig. 5d), indicating that G-1 can significantly inhibit the activation of NF-κB in CRC cells. To investigate whether downregulation of NF-κB is involved in G-1 induced growth arrest of CRC cells, we treated HCT-116 and SW480 cells with NF-κB inhibitor BAY11-7082. The results showed that BAY11-7082 can also suppress the proliferation of both HCT-116 and SW480 cells (Fig. 5e). Further, we overexpressed p65 in HCT-116 and SW480 cells by transfection with pcDNA3.1/p65 plasmid (Additional file 1: Figure S4), which revealed that overexpression of p65 can significantly reverse the growth arrest effects of G-1 in both HCT-116 and SW480 cells (Fig. 5f). These data suggested that inhibition of NF-κB participated G-1 induced growth arrest of CRC cells.

Next we examined the mechanisms of G-1 induced suppression of NF-κB. Our data revealed that G-1 treatment can suppress the phosphorylation of IκBα since treatment for 30 min (Fig. 5g). It might be due to that G-1 treatment can inhibit the activation of IKKα (Fig. 5g), which is a kinase can phosphorylate IκBα and then lead it to degradation. This is confirmed that G-1 treatment resulted in the suppression of mRNA expression of IKKα, while not IKKβ or IKKγ (Fig. 5h). In addition, our data showed that G-1 can increase the phosphorylation of GSK-3β (Fig. 5i) and its association with p65 (Fig. 5j), which will maintain an inactive state and negative control the activation of NF-κB [30]. Collectively, the results suggested that both canonical IKKα/IκBα pathways and phosphorylation of GSK-3β were involved in G-1 induced inhibition of NF-κB in CRC cells.

### Activation of GPER suppressed the progression of CRC in vivo

**Fig. 6:**
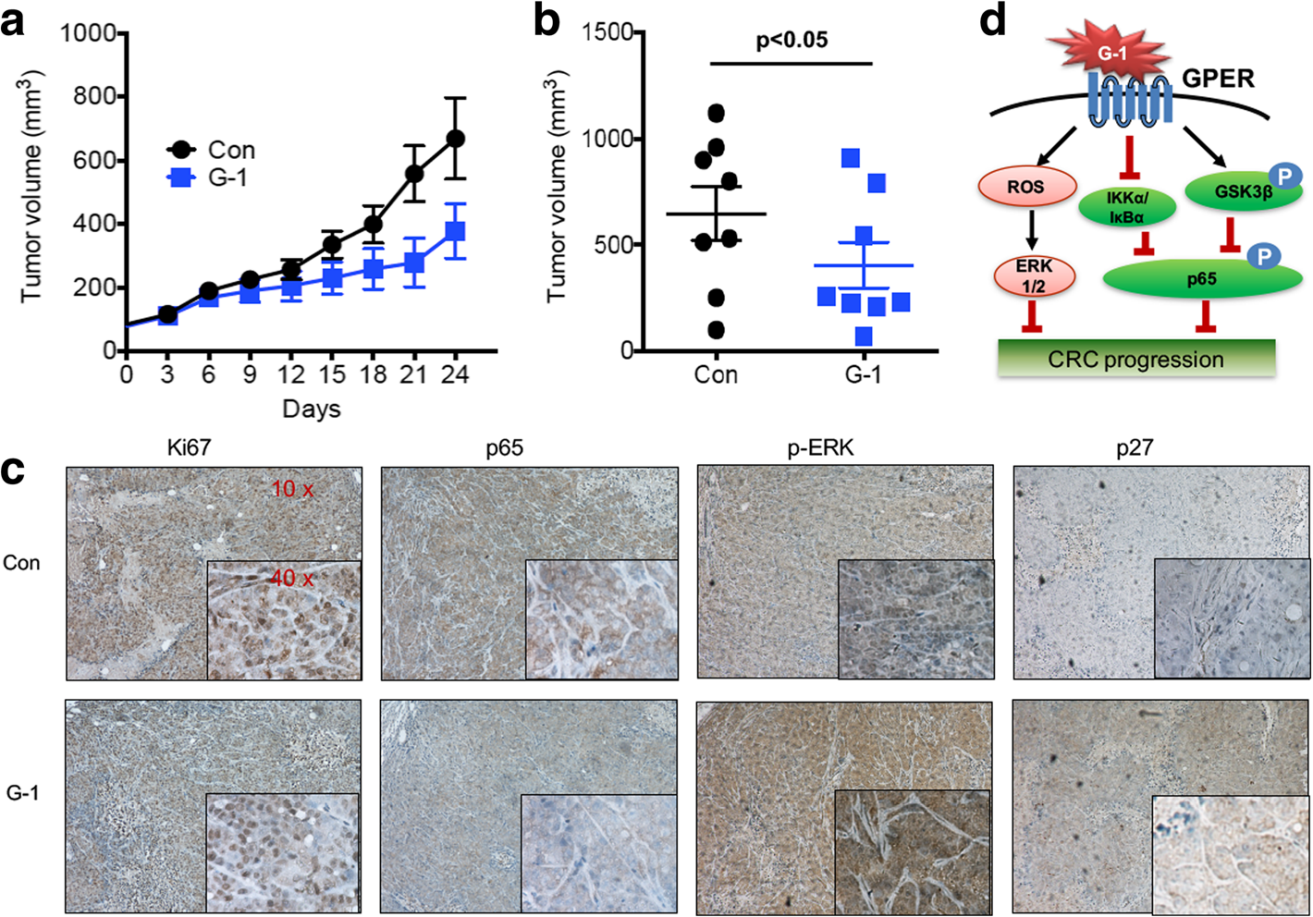
Activation of GPER suppressed the in vivo progression of CRC. **a** The tumor volumes of G-1 and control group were measured in HCT-116 xenograft models at the indicated time interval; **b** The tumor volumes of G-1 and control group at the end of experiment; **c** The tumor tissue sections of G-1 and control group of HCT-116 xenograft models were subjected to IHC detection of Ki-67, p65, p-ERK1/2, and p27; **d** A proposed model to illustrate the mechanisms of GPER mediated growth suppression of CRC cells

To further study the regulatory effects of GPER on the tumor progression in vivo, we examined the effect of G-1 on progression of HCT-116 tumor xenografts in nude mice. We found that G-1 treatment markedly inhibited tumor growth of HCT-116 cells in vivo (Fig. 6a&b). Additionally, we used the monoclonal antibody Ki67, which recognizes a nuclear antigen expressed by proliferating cells, to detect proliferating cells in tumor. Decreased Ki67-positive cells in G-1 group were also detected in vivo (Fig. 6c). Further, the results of immunohistochemistry confirmed that G-1 treatment can decrease the expression of p65 while elevate the expression of p-ERK1/2 and p27 (Fig. 6c). These data suggested that activation of GPER can suppress the growth of CRC in vivo via activation of ERK1/2 and suppression of NF-κB.

## Discussion

Although estrogenic signals have been suggested to modulate the tumorigenesis and progression of CRC, the roles and mechanisms of GPER, a novel membrane estrogen receptor, in the development of CRC have never been investigated. Our present study revealed that the expression of GPER in tumor tissues were markedly less than that in their correspondingly adjacent normal mucosa tissues. Patients whose tumors expressing less GPER showed significant (*p* < 0.01) poorer survival rate as compared with those with greater levels of GPER. Promoter methylation and histone acetylation were involved in down regulation of GPER in CRC cell and tissues. Activation of GPER by its specific agonist G-1 can suppress the proliferation, induce G2/M phase arrest, elevate ER stress, and increase the mitochondrial-related apoptosis in CRC cells. ROS/ERK1/2 and inhibition of NF-κB were involved in G-1 induced growth suppression. Both canonical IKKα/ IκBα pathways and phosphorylation of GSK-3β were involved in G-1 induced inhibition of NF-κB in CRC cells. Collectively, activation of GPER can inhibit the growth of CRC cells both in vitro and in vivo through multiple intracellular signaling pathways as summarized in Fig. 6d.

Our data revealed for the first time that GPER might be a potential valuable target for CRC therapy. A large body of evidences from preclinical studies suggested that estrogenic signals regulate the progression of CRC, which was evidenced by the factor that women have a lower risk for CRC than man and exposure to estrogen can decrease the CRC risks [10]. Previous studies indicated that ERβ is a target for CRC prevention due to its activation reduced intestinal tumor formation and represses oncogenes, while its knockout mice exhibited changes in colonic epithelia [10]. As a recently identified membrane estrogenic receptor, more and more evidences supporting GPER acts as a tumor-suppressor [31]. Our previous studies and published literatures indicated that activation of GPER by its specific agonist G-1 can suppress the progression of various cancers including Leydig [32], prostate [33], ovarian [31], and breast [18, 23] cancer. Our present study showed that G-1 can suppress in vitro proliferation of CRC cells via induction of G2/M phase arrest, ER stress and mitochondrial related apoptosis. This was supported by in vivo data that G-1 treatment inhibited growth of HCT-116 xenograft tumor in nude mice. Therefore GPER might be involved in the protective roles of estrogenic signals on CRC development because it can be activated by E2 in human body [34] and therefore inhibits the cancer progression. Some other studies suggested that GPER activation can trigger the growth and progression of breast [35] and endometrial [36] cancer cells. As previously discussed [18, 23], it might be due to the specificities of agonist and difference of cell types and treatment conditions.

Our data showed that GPER expression was down regulated in CRC tissues as compared with the paired adjacent normal tissues. In addition, patients whose tumors expressing less GPER showed significant (*p* < 0.01) poorer survival rate as compared with those with greater levels of GPER. Promoter methylation and histone acetylation were involved in the down regulation of GPER in CRC cell and tissues. Similar results also were observed in ovarian [31] and breast [23] cancer, which showed that GPER expression was significantly lower in cancer tissue than in benign and low-malignant tumors or the paired normal tissues. The greater expression of GPER was associated with a longer recurrent-free survival (RFS) in breast cancer patients [37]. Other studies also revealed that GPER expression had no correlation with clinical outcome [38] or even was a marker to predict poor survival [39] of cancer patients. The inconsistent observations might be due to variations of cancer types and subcellular localization of GPER [24]. Epigenetic mechanism of promoter methylation was involved in the low expression of GPER in CRC tissue and cell lines. This is consistent with the results in breast cancer that GPER expression is inactivated by promoter methylation in breast cancer cell lines and primary breast cancer tissue derived from patients, while inactivation of DNA-methyltransferase by 5-Aza increased GPER expression [40]. In addition, our data firstly revealed that H3 acetylation also played a critical role in the regulation of GPER expression in CRC. It suggested that epigenetic suppression might be a general mechanism for the down regulation of GPER among different cancers.

As summarized in Fig. 6d, ROS/ERK1/2 and inhibition of NF-κB were involved in G-1 induced growth suppression of CRC. ROS generation mediated a wide ranges of cellular responses such as growth arrest and apoptosis [41]. Cellular accumulation of ROS caused sustained ERK1/2 activation can lead to cell death [42]. Similarly, G-1 induced the ROS generation of acidophilic granulocyte [43], cardiac myocytes [44], and breast cancer cells [18]. Both NAC (ROS scavenger) and PD98059 (ERK1/2) can attenuate G-1 induced proliferation inhibition of CRC cells in the present study. As revealed in our previous studies performed in breast cancer cells [23], G-1 can also suppress the activity of NF-κB in CRC cells via increasing the phosphorylation of GSK-3β and its association with p65. The phosphorylated GSK-3β, which was maintained an inactive state, can negative control the activation of NF-κB and cell proliferation [30]. Beyond that, our present study revealed for the first time that G-1 can also inhibit NF-κB via decreasing the mRNA expression and protein phosphorylation of IKKα and then suppressing IκBα. This indicated that canonical IKKα/ IκBα pathways was also involved in G-1 induced suppression of NF-κB. While the mechanisms of G-1 induced IKKα inhibition need further research.

## Conclusions

Our results indicate that GPER functions as a tumor suppressor in CRC via activation of ROS/ERK1/2 and suppression of NF-κB. Taken together with published literatures, these findsings suggested that GPER is an important target and G-1 is a drug candidate for CRC therapy.

## Additional file

Additional file 1: Figure S1. GPER expression was down regulated in male CRC patients. The relative mRNA expression of GPER in TCGA Colorectal patients from Oncomine datasets with 117 males and 120 females. **Figure S2.** G-1 treatment induced apoptosis and ER stress. (A) SW480 cells were treated with increasing concentrations of G-1 for 48 h, stained with annexin V-FITC and PI, and then analyzed by flow cytometry for cell apoptosis; (B) HCT-116 cells were treated with G-1 as the indicated concentrations for 48 h, and then Bcl-2, Bax, caspase3, and p21 protein expression levels were analyzed by Western-blot analysis; (C) HCT-116 cells were treated with 1 μM G-1 for the indicated times, and then the expression of ATF4, ATF6, XBP-1 and CHOP were determined by Western-blot analysis. **Figure S3.** Effects of G-1 on activation of MAPK signals in HCT-116 cells. HCT-116 cells were treated with increasing concentrations of G-1 for 30 min (A) or 1 μM G-1 for the indicated times (B), the total and phosphorylation of MAPK were measured by Western blot analysis. **Figure S4.** Over expression of p65 in CRC cells. HCT-116 and SW480 cells were transfected with pcDNA3.1 (vector) or pcDNA/p65 for 24 h, the protein expression of p65 was measured by use of Western blot analysis. **Table S1.** The detailed clinicopathological features of clinical CRC tissues of Cohort 1 (*n* = 32). (DOCX 1759 kb)
