# Supplementary material for: Epigenetic down regulation of G protein-coupled estrogen receptor (GPER) functions as a tumor suppressor in colorectal cancer
Source: Mol Cancer. 2017 May 5;16:87. doi: 10.1186/s12943-017-0654-3 (PMC5418684; doi:10.1186/s12943-017-0654-3)
Supplement: Additional file 1: Figure S1. — GPER expression was down regulated in male CRC patients. The relative mRNA expression of GPER in TCGA Colorectal patients from Oncomine datasets with 117 males and 120 females. Figure S2. G-1 treatment induced apoptosis and ER stress. (A) SW480 cells were treated with increasing concentrations of G-1 for 48 h, stained with annexin V-FITC and PI, and then analyzed by flow cytometry for cell apoptosis; (B) HCT-116 cells were treated with G-1 as the indicated concentrations for 48 h, and then Bcl-2, Bax, caspase3, and p21 protein expression levels were analyzed by Western-blot analysis; (C) HCT-116 cells were treated with 1 μM G-1 for the indicated times, and then the expression of ATF4, ATF6, XBP-1 and CHOP were determined by Western-blot analysis. Figure S3. Effects of G-1 on activation of MAPK signals in HCT-116 cells. HCT-116 cells were treated with increasing concentrations of G-1 for 30 min (A) or 1 μM G-1 for the indicated times (B), the total and phosphorylation of MAPK were measured by Western blot analysis. Figure S4. Over expression of p65 in CRC cells. HCT-116 and SW480 cells were transfected with pcDNA3.1 (vector) or pcDNA/p65 for 24 h, the protein expression of p65 was measured by use of Western blot analysis. Table S1. The detailed clinicopathological features of clinical CRC tissues of Cohort 1 (n = 32). (DOCX 1759 kb) [file 12943_2017_654_MOESM1_ESM.docx]

**Supplementary data for**

**Epigenetic down regulation of G protein-coupled estrogen receptor (GPER) functions as a tumor suppressor in colorectal cancer**

Qiao Liu^1#^, Zhuo-Jia Chen^2#^, Guan-Min Jiang^3^, Yan Zhou^1^, Xiang-Ling Yang^4^, Hong-Bin Huang^2^, Huan-Liang Liu^4^, Jun Du^1^, Hong-Sheng Wang^1*^

*^1^ Department of Microbial and Biochemical Pharmacy, School of Pharmaceutical Sciences, Sun Yat-sen University, Guangzhou 510006, China;*

*^2^* *Sun Yat-sen University Cancer Center; State Key Laboratory of Oncology in South China; Collaborative Innovation Center for Cancer Medicine, Guangzhou 510060, China;*

*^3^ Hunan Cancer Hospital & The Affiliated Cancer Hospital of Xiangya School of Medicine, Central South University, Changsha 410013, China*

*^4^ Guangdong Institute of Gastroenterology and the Sixth Affiliated Hospital, Institute of Human Virology, Key Laboratory of Tropical Disease Control (Ministry of Education), Sun Yat-sen University, Guangzhou 510655, China*

^#^ These authors contributed equally to this work.

*** Corresponding Authors:** Dr HS Wang Email: [whongsh@mail.sysu.edu.cn](mailto:whongsh@mail.sysu.edu.cn); [hongshengwang@foxmail.com](mailto:hongshengwang@foxmail.com), Department of Microbial and Biochemical Pharmacy, School of Pharmaceutical Sciences, Sun Yat-sen University, Guangzhou 510006, China.

***Flow cytometry***

For cell cycle analysis, cells were synchronized at the G1/S transition by a double TdR block, as follows: 16 h block with 2.5μM TdR (Sigma), 10 h release followed by the second block for 16 h. Then cells were treated with or without G-1, washed with PBS, fixed with 70% ethanol overnight at 4 °C, incubated with propidium iodide (PI, 50μg/mL, Sigma), and analyzed by a Coulter Epics XL Flow Cytometry System (Beckman-Coulter, Miami, USA). Cell cycle analysis was performed using CELL Quest program (Becton Dickinson). For cell apoptosis analysis, both the suspension and the adherent cells were collected after G-1 treatment, stained with Annexin V-FITC for 15 min and propidium iodide for 5 min, and analyzed immediately by flow cytometry. For ∆Ψm measurement, JC-1 staining solution (5μg/ml) was added to G-1 treated cells for 20min. After washed with PBS twice, mitochondrial membrane potentials were monitored by determining the relative amounts of dual emission from a multiple fluorescence reader. ROS were monitored with the oxidation sensitive fluorescent probe 2’7’-dichlorodihydrofluorescein diacetate (DCF-DA) according to our previous methods ([Ge, Chen et al. 2014](#_ENREF_9)).

***Western blot analysis and immunoprecipitation***

Western blot analysis was performed as previously described ([Jiang, Wang et al. 2013](#_ENREF_13)). Briefly, cells were lysed in cell lysis buffer, and then lysates were cleared by centrifugation and denatured by boiling in Laemmli buffer. Protein concentration was measured using the Bio-Rad protein assay kit. Approximately 20 μg protein were separated on 10% SDS-PAGE and electrophoretically transferred to nitrocellulose membranes. Following blocking with 5% non-fat milk at room temperature for 2 h, membranes were washed for three times with PBS, incubated with the primary antibody at 1:1000 dilution overnight at 4 °C and then incubated with a horseradish peroxidase-conjugated secondary antibody at 1:5000 dilution for 2 h at room temperature, and detected with the Western Lightning Chemiluminescent detection reagent (Perkin-Elmer Life Sciences, Wellesley, MA).

For immunoprecipitation analysis, cells were washed three time with ice-cold PBS and harvested at 4 ℃ in immunoprecipitation lysis buffer containing 50mM HEPES, pH 7.5, 150mM NaCl, 0.5% NP-40, 2mM EDTA, 10% glycerol, 1mM Na_3_VO_4_, 1mM NaF, 1mM dithiothreitol, 1mM 4-(2-aminoethyl) benzenesulfonyl fluoride,1 μg/ml leupeptin, 1 μg/ml pepstatin and 1μg/ml aprotinin. Equal amounts of protein were immunoprecipitated using anti-p65 antibody, and the immune complexes were bound to protein A/G Sepharose. The beads were washed with lysis buffer and subjected to western blotting with anti- GSK-3β antibody.

***Immunofluorescence***

Immunofluorescent staining was carried out as described previously (Wei et al., 2014). Briefly, cells were cultured on chamber slides, serum starved for 12 h, then exposed to G-1 for the indicated time periods. Cells were washed three times with PBS, fixed with 4% paraformaldehyde for 20 min and permeabilized with 0.3% Triton X-100 for 10 min. After blocking with goat serum for 2 h at room temperature, cells were incubated with antibodies against p65 (1:100 dilutions) at 4 °C overnight. Slides were washed three times with PBS and incubated with Alexa Fluor 488-conjugated secondary antibodies (1:1000 dilutions) for 1 h at room temperature. Nuclei were stained with DAPI for 10 min. Samples were examined with Confocal Laser Scanning Microscopy (Zeiss) to analyze expression of p65.

**Figure S1**

**Figure S1 GPER expression was down regulated in male CRC patients*.*** The relative mRNA expression of GPER in TCGA Colorectal patients from Oncomine datasets with 117 males and 120 females.

**Figure S2**

**Figure S2 G-1 treatment induced apoptosis and ER stress.** (A) SW480 cells were treated with increasing concentrations of G-1 for 48h, stained with annexin V-FITC and PI, and then analyzed by flow cytometry for cell apoptosis; (B) HCT-116 cells were treated with G-1 as the indicated concentrations for 48 h, and then Bcl-2, Bax, caspase3, and p21 protein expression levels were analyzed by Western-blot analysis; (C) HCT-116 cells were treated with 1μM G-1 for the indicated times, and then the expression of ATF4, ATF6, XBP-1 and CHOP were determined by Western-blot analysis.

**Figure S3**

**Figure S3 Effects of G-1 on activation of MAPK signals in HCT-116 cells.** HCT-116 cells were treated with increasing concentrations of G-1 for 30min (A) or 1 μM G-1 for the indicated times (B), the total and phosphorylation of MAPK were measured by Western blot analysis.

**Figure S4**

**Figure S4 Over expression of p65 in CRC cells**. HCT-116 and SW480 cells were transfected with pcDNA3.1 (vector) or pcDNA/p65 for 24 h, the protein expression of p65 was measured by use of Western blot analysis.

**Table S1 The detailed clinicopathological features of clinical CRC tissues of Cohort 1 (n=32).**

| **Characteristics** | | **N** | **p value** |
| --- | --- | --- | --- |
| Tumor/Adjacent | Tumor | 32 | <0.01 |
|  | Adjacent | 32 |  |
| Age | ≤50 | 11 | 0.802 |
|  | ＞50 | 21 |  |
| Sex | Male | 11 | 0.468 |
|  | Female | 21 |  |
| Stage | 1 | 4 | 0.254 |
|  | 2 | 14 |  |
|  | 3 | 10 |  |
|  | 4 | 4 |  |
